# Gambling Outlets Location in Urban Areas: A Case Study of Madrid

**DOI:** 10.1007/s10899-021-10094-3

**Published:** 2021-12-08

**Authors:** Levi Pérez, Ana Rodríguez, Andrey Shmarev

**Affiliations:** 1 Department of Economics, Jovellanos Faculty of Commerce, Tourism and Social Sciences, University of Oviedo, Luis Moya Blanco 261, 33203 Gijón, Spain; 2 Department of Economics, School of Economics and Business, University of Oviedo, Av. del Cristo, sn, 33006 Oviedo, Spain

**Keywords:** Gambling outlets, Cities, Neighborhoods, Co-location, Spatial econometric analysis

## Abstract

Cities are certainly a key factor in the location of gambling facilities. This paper aims to map the location of gambling outlets in urban areas and to examine potential links between neighborhoods socioeconomic and demographic characteristics and gambling supply, taking into account spatial dependencies of neighboring areas. This correlation is of interest because neighborhood characteristics may attract sellers, and because the presence of gambling sellers may cause changes in neighborhood demographics. Using detailed official data from the city of Madrid for the year 2017, three spatial econometric approaches are considered: spatial autoregressive (SAR) model, spatial error model (SEM) and spatial lag of X (explicative variables) model (SLX). Empirical analysis finds a strong correlation between neighborhoods characteristics and co-location of gambling outlets, highlighting a specific geographic patterning of distribution within more disadvantaged urban areas. This may have interesting implications for gambling stakeholders and for local governments when it comes to the introduction and/or increase of gambling availability.

## Introduction

Commercial gambling opportunities have greatly expanded throughout many jurisdictions worldwide in recent years. However, the scope of this phenomenon differs across countries showing an overall recent decrease gambling participation but a significant increase in vulnerable population sectors (Abbott et al., 2014; Welte et al., 2015). Even though the “availability hypothesis” suggests that a positive correlation exists between gambling participation and expenditure and the number of opportunities to gamble (Orford, 2002; Storer et al., 2009), participation in such activities may be conditioned not only by the availability and exposure of gambling, which are ultimately determined by many institutional factors, such as the main regulatory policies, but also by the willingness of individuals to gamble—in fact, if consumers prefer a corner solution (that is, they choose not to gamble), an expansion of gambling opportunities will have limited effect on consumer's behavior (Kearney, 2005). While gambling operators and firms are basically interested in earning positive profits, local governments may be influenced by the characteristics of their own jurisdiction and those of neighboring areas (Wenz, 2008). Where gambling is allowed, governments have traded its negative aspects for the potential benefits—tax revenues, jobs, and other economic development initiatives… -; as the driving for legalization and regulation (Nichols & Tosun, 2017).

Skidmore and Tosun (2008) found that the introduction of gambling products within a jurisdiction can have an impact on retail activity, suggesting that some economic benefits result from opening new gambling businesses. As for the negative side, expansion of gambling opportunities to an area could raise social concerns linked to a number of negative externalities, including regressivity of gambling taxation (Gandullia & Leporatti, 2018; Perez & Humphreys, 2011), public health impacts (Wardle et al., 2014) and gambling-related harm beyond the loss of money (pathological gambling, social life and health issues, work performance, crime…) (Delfabbro & King, 2019; Grinols & Mustard, 2001). In addition, gambling could also be considered as immoral (Basham & White, 2002).

All these possible effects of exposure and accessibility to gambling opportunities exhibit a certain social and geographical patterning. In fact, previous research has explored the distribution of gambling outlets (Robitaille & Herjean, 2008; Wardle et al., 2014) and it has recognized the role environment plays in the relationship between access to gambling opportunities and individuals’ behavior (Korn & Shaffer, 1999; Pearce et al., 2008). In general, analyses of spatial distribution of gambling show that people living in the most disadvantaged areas have greater access to gambling and are more affected by the harms of gambling (Papineau et al., 2020). The links between gambling availability and area characteristics, such as socioeconomic environment, have also been explored by Gilliland and Ross (2005). In addition, Beckert and Lutter (2009) explain that the lack of leisure opportunities for socially disadvantage people contributes to the expansion of gambling. Finally, it has been reported that an increased availability and accessibility of gambling outlets is related to an increase in related unhealthy behaviors and increased likelihood of problem gambling (Pearce et al., 2008; Rush et al., 2007; Young et al., 2012), with those living in areas of greater deprivation being more likely to experience harm (Orford et al., 2010).

As for measuring area-level socio-economic status most previous studies have considered information about the areas’ degree of education, age structure of the population, income of households and unemployment rate (Raisamo et al., 2019). Along with sociodemographic variables, other studies, including Carrà et al. (2017), who analyses associations between gambling and baseline individual and area-level characteristics, and Marek et al. (2021), that examines how ‘environmental goods’ such as green spaces and ‘environmental bads’ such as alcohol outlets and gaming venues co-occur, used composite index at small level area to measure area-level deprivation. Even in that cases, variables such income, employment and education are considered as domains of relative deprivation (alongside other indicators such as health deprivation and disability, barriers to housing and services, crime and disorder, and living environment).

Cities are certainly a key factor in the location of gambling (Fiedor et al., 2017). Spatially, gambling is concentrated mainly in cities and large urban agglomerations (Klebanow & Gallaway, 2015), which contributes to create a specific retail environment and shaping the urban (Markham, 2015). Marshall (2009) highlighted the role of the local environment as a key determinant of the gambling intensity, while O’Flaherty and Sethi (2010) documented that street vice activities (including gambling) are largely limited to neighborhoods that are centrally located and densely populated. Indeed, recent studies have put attention on gambling environments in cities when addressing social concerns with respect to gambling exposure. Papineau et al. (2020) characterize gambling environments in Quebec (Canada), Espadafor and Martínez (2021) estimate the effect of gambling opportunities on educational performance in Madrid (Spain), and Macdonald et al. (2018) examine the socio-spatial patterning of outlets such as alcohol, fast food, tobacco and gambling, within Glasgow City (Scotland).

In this paper the focus is on the relationship of the gambling retail environment with urban area (neighborhood) characteristics. This correlation is of interest because neighborhood characteristics may attract sellers, and because the presence of gambling sellers may cause changes in neighborhood demographics. This leads to the question why gambling opportunities concentrate in some neighborhoods. Additionally, it is claimed that people in deprived areas are more likely to gamble and that gambling outlets clusters are associated with higher rates of problems among individuals from lower socio-economic groups (Abbott et al., 2004; Livingstone, 2001; Wheeler et al., 2006). As in Grumstrup and Nichols (2021), it is argued that the concentration of gambling outlets can be mostly explained by income and other neighborhood characteristics.

In particular, the empirical exercise examines whether certain urban areas are subject to excess access to gambling retailers. Specifically, it aims to explain how the number of gambling outlets located in a certain neighborhood correlates with income and other sociodemographic characteristics of that local area, as previously mentioned, but taking into account the supply of nearby areas. To ensure consistent and efficient estimates, the estimate model of gambling location tests and corrects for spatial effects. As discussed in Garrett and Marsh (2002), among others, spatial dependence results from a lack of independence among cross-sectional units caused, among others, by the presence of direct influence of neighboring units.

The focus is on Madrid (Spain), where, as far as it is known, there has been no evaluation of the distribution of gambling opportunities and their spatial patterning. This is an interesting case of study since the Spanish gambling market has seen a dramatic increase in both economic figures and opportunities over the last decades. Until 1977, legal gambling was severely restricted and non-legal gambling mostly criminalized. Then, first licenses to privately operate casino gambling, bingo and slots machines were awarded and these types of establishments became more common in the main Spanish cities. In 2008, several bookmakers were awarded the first licenses to operate in Madrid, the first jurisdiction in Spain in allowing offline sports betting.

Following this practice, other Spanish regions also allowed bookmakers to operate, setting up a completely new gambling market and urban retail landscape. Indeed, the city of Madrid has experienced a significant increase in the supply of gambling opportunities in recent years reaching more than 800 gambling outlets at the end of 2017. As in Espadafor and Martínez (2021), the choice of Madrid as case study is then motivated by the intensive spread of new gambling outlets between 2015 and 2017. Was this increase in the access to gambling spatially uniform or was there a trend towards deprived neighborhoods? Is there a spillover effect at the neighborhood level or is the gambling supply dependent exclusively on the own determinants of the area? These are some of the questions this paper endeavors to answer by trying add to the increasing social debate about the perceived clustering of gambling opportunities in areas of greatest socio-economic deprivation.

The next section describes the methods and provides some background on the spread of gambling opportunities in Madrid. Later, the results are discussed, followed by the conclusions section which contains concluding remarks suggesting that gambling opportunities display similar spatial patterning across urban areas.

## Materials and Methods

Using official municipal data from the city of Madrid for the year 2017 and various spatial regression techniques, we examine the potential links between neighborhoods’ socioeconomic and demographic characteristics and gambling retail stores.

### Sample: A Case Study of Madrid

Madrid is the largest city of Spain. The city has a metropolitan area population exceeding 6.5 million. It includes 21 districts comprising 131 neighborhoods. The most populated neighborhood is *Aluche* (in *Latina* district) with almost 66,000 inhabitants in 2017, while the least populated area is located in *El Cañaveral* neighborhood (in *Vicálvaro* district) with just 945 inhabitants. The average population of the neighborhoods of the city of Madrid is 24,594 inhabitants (standard deviation is 13,624) while the average rate of migrant population (non-native) is 12.6—with half of the neighborhoods accounting for almost 80% of it. The highest density of the population in the city of Madrid is in *Gaztambide* (in *Chamberí* district) with 448 inhabitants per hectare.

Also, in terms of income, neighborhoods are not uniform. The 10% of the neighborhoods accumulate 20% of the total city income.

### Gambling Outlets Data Collection

The City Council of Madrid (*Ayuntamiento de Madrid*) is the body responsible for the government and administration of the municipality. In 2008, Spain’s first sports betting shops were allowed to open in Madrid. As an immediate outcome, some gambling firms planned to set up 70 sports betting shops across the Madrid area—the first jurisdiction in Spain in granting licenses. Within this context, the number of privately-operated licensed gambling outlets in the city of Madrid increased from 304 in 2013 to 509 in 2017. Also considering the number of lottery stores operated by either by SELAE (a state-owned company responsible for the operation of all types of lotteries) or ONCE (the national organization of Spanish blind people which is awarded a license to operate a charity lottery), total number of gambling outlets located in Madrid reached 812 in 2017. However, the gambling landscape in the city of Madrid does not draw an equally distributed map. The neighborhoods of *Vista Alegre* and *Embajadores* host more than 20 gambling outlets each, while there are 10 neighborhoods with none. Almost 50% of all gambling outlets are concentrated in 29 neighborhoods (out of 131) – each of them hosting at least 10 or more gambling outlets. There are 15 neighborhoods with just one gambling outlet.

Address data for gambling outlets as for 2017 were obtained from the Madrid City Council Open Data (i.e. the open data web site of the City Council of Madrid) according to the National Classification of Economic Activities (CNAE 2009) which listed all existing and operating businesses within the city of Madrid. Considering this administrative classification, the category that is of interest to this paper comprises “*all venues destined to satisfying the gambling needs of the public including casino gambling, bingo and gambling halls as well as horse betting and lotto*”– which includes, as previously mentioned, a total of 812 gambling outlets. The data held is deemed as comprehensive as information on the various premises is required to be held by Madrid City Council for inspection, planning and licensing purposes. As in Macdonald et al. (2018), the address for the outlets were linked to precise geo-coordinates via the Madrid City Council Open Data and then assigned to a specific neighborhood accordingly. So the dependent variable in the spatial model is the number of gambling outlets located in a certain neighborhood.

### Measures: Covariates and Controls

Measures describing the neighborhood-level socio-economic characteristics were based on data from the Madrid City Council Open Data. The data is based on open data containing information about the neighborhoods’ population, population density—the number of people per hectare -, the rate of native Spanish population, age structure of the population—so the number of people aged 0 to 15 divided by the sum of people 16 to 65 and 65 and over—to proxy the socio-demographic structure of each neighborhood’s population, the mean of household income in Euros as for 2016; and the unemployment rate. The data used was available at the neighborhood level. Madrid neighborhoods areas are obviously defined for a different purpose, and they are not coherent communities and vary in population and size. Notwithstanding, neighborhoods should reveal the socio-economic exposure to gambling outlets in daily life reasonably well. Table 1 shows the descriptive statistics for the considered covariates which encompass socioeconomic and demographic indicators for each of the neighborhoods of the city of Madrid.

**Table 1:** Descriptive statistics

Variable	Mean	SD	Min	Max
Gambling outlets (number)	6.27	5.06	0.00	24.00
Population (hab.)	24,594.08	13,571.91	945	65,961
Population density (hab./hectare)	181.02	119.75	0.20	448.00
Native Spanish population rate (%)	87.42	5.90	68.07	96.74
Youth population rate (%)	17.57	7.10	7.56	49.87
Income (euros)	41,746.00	16,599.00	19,674.00	89,015.00
Unemployment rate (%)	7.88	2.63	3.20	15.04

131 observations

### Data Analysis

**Fig. 1:**
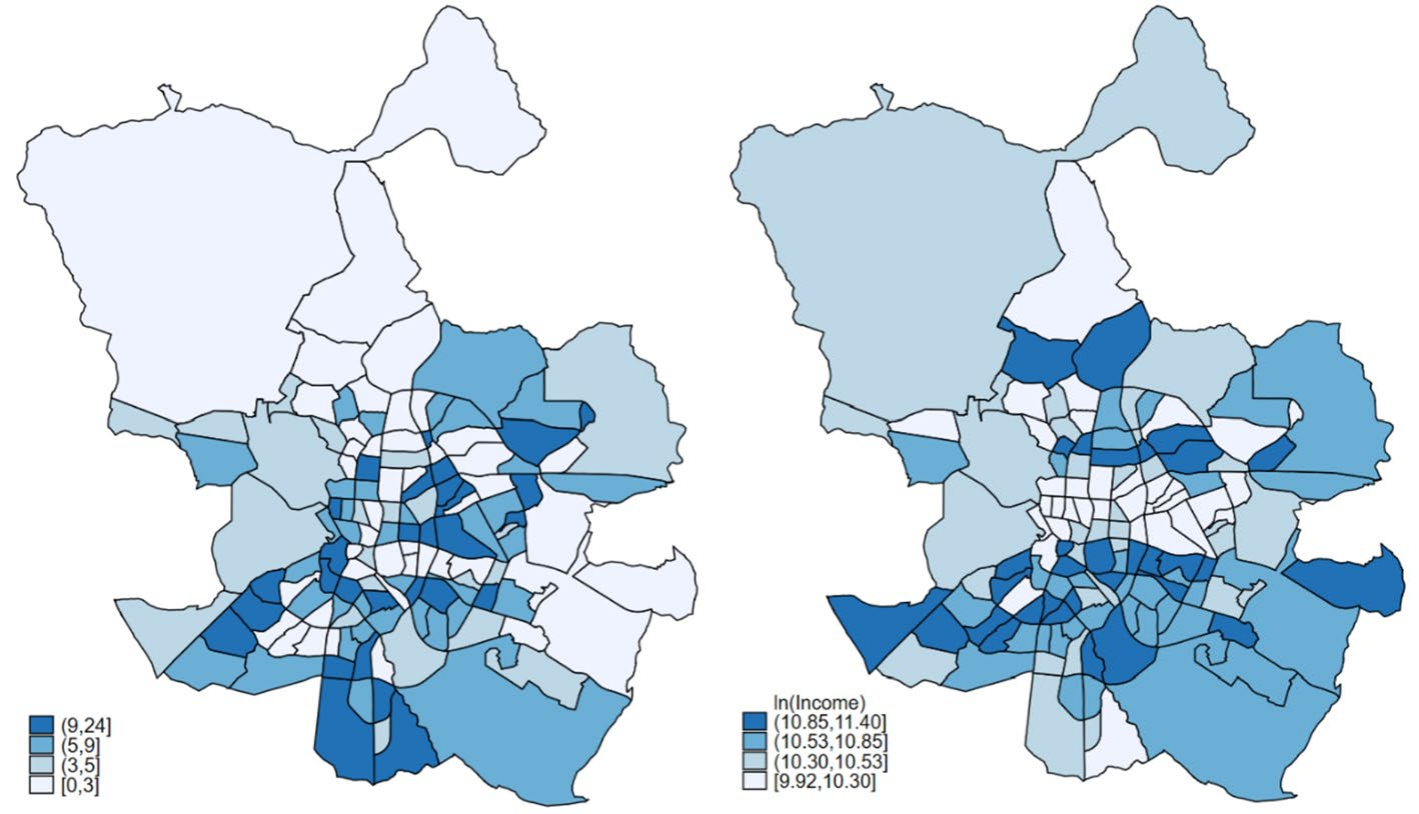
Gambling outlets (left) and income (right) (neighborhoods of the city of Madrid. 2017)

On the Fig. 1 map, the distribution of gambling outlets and household income level across neighborhoods are shown, where darker colors indicate higher number of gambling outlets and larger level of income respectively; each category represents a quantile. A potential positive spatial autocorrelation in these data can be observed which can be described by the first law of geography which states that near things are stronger related than distant things (Tobler, 1970). First, a district with a high number of gambling outlets is surrounded by other districts with also a significant number of this type of venues. In order to determine whether there are significant spatial associations in the data, we use the widely used Moran’s I test. This test is used to assess spatial dependence in the outcome from a model. Positive values for Moran’s I indicate that similar units are near one another (that is, *positive* spatial autocorrelation)—see for example Elhorst (2014) for further details. To undertake a Moran’s I test, we need to first specify how the units are related, that is, provide a W matrix. In this sense, we use a queen geographic contiguity (normalized by row) matrix. Using this W, we test for spatial autocorrelation in the data using Moran’s I. Focusing first on the outcome, we find statistically significant spatial autocorrelation (chi2(1) = 4.51; Prob. > chi2 = 0.03), which confirms the preliminary insights from Fig. 1 that gambling outlets are not entirely random across the city. This can explain by a variety of spatial processes and mechanisms including neighborhoods’ socioeconomic and demographic characteristics may cause gambling outlets to cluster, endogenous interaction effects (the number of gambling outlets in a neighborhood can affect another gambling business decisions).

In addition, due to the maturity of the Spanish gambling market in 2017, it could be assumed that rent-seeking agents have taken into consideration which location to occupy based on their decisions. That is to say, we are not assuming independence of the variable Y between spatial units is not assumed. The operators would not establish new gambling outlets if the market is too crowded and would reasonably target spaces where the competition would be lower.

Second, a negative correlation can be primarily observed between household income and gambling outlets. However, by deeper analyzing this relationship, it is necessary to address a potential endogeneity problem, given that the relation between both variables could be bidirectional. This issue is tackled in the model considering values of the income variable lagged one period (so as predetermined).

### Spatial Econometric Models

In order to model spatial autocorrelation in a way that other covariates are included in the analysis three econometric approaches are considered: spatial autoregressive (SAR) model, spatial error model (SEM) and spatial lag of X (explicative variables) model (SLX). Spatial econometric models allow to address heterogeneities across observations and assess spatial autocorrelation. In the spatial econometric framework, spatial dependence assumes that values observed for one area depend on the values of neighboring observations at nearby areas and vice versa (LeSage & Pace, 2009).

The standard approach with spatial econometric models would be to establish a benchmark model that needs to be expanded with spatial interaction effects (Hendry, 1995). With this aim, we start with a non-spatial linear OLS regression:

1 $$Y = X\beta + \varepsilon$$

where the dependent variable Y will be an N X 1 vector denoted as the number of gambling outlets in a spatial observation unit (here, a neighborhood); X will be the N x K matrix of exogenous explanatory variables; $$\beta$$ the K × 1 vector of parameters to be estimated and $$\varepsilon$$ the i.i.d disturbance term vector, $$\varepsilon$$$$\approx{ }iid{ }N\left( {0,\sigma_{{}}^{2} } \right){ }$$.

As explained above, the proposed model accounts for the existence of unobservable heterogeneity within the distribution of the gambling outlets across the different neighborhoods within the city of Madrid. To account for this situation, a spatial econometric model that assumes an underlying spatial autoregressive process is proposed, either in the interactions of the dependent variable of the neighborhood j with the dependent variables of its neighbors (SAR model); the interactions of the error terms amongst themselves (SEM model); and including lagged independent variables from neighboring spatial units (SLX model). Certainly, it is also possible to define other spatial models, for example, controlling for both the interactions between the independent variables and the error terms at the same time (SAC model)—see Elhorst (2014), among others, for further details on this.

Once the weights matrix W is defined, the specification of the model in Eq. 1 is updated with an WY indicator:

2 $$Y = \rho WY + X\beta + \varepsilon$$

where the $\rho$ (spatial autoregressive coefficient) accounts for the impact of the dependent variable of nearby neighborhoods. This is a first order autoregressive process in which $\rho$ will range between − 1 and 1 (Elhorst, 2014).

Equation 2 corresponds to the spatial autoregressive (SAR) model which assumes that the unobservable heterogeneity is captured solely by the dependent variable Y. The spatial autoregressive coefficient will indicate whether the presence of nearby gambling outlets will impact the decision of establishing a new venue while controlling for exogenous covariates X.

The second spatial econometric model considered in this paper is the spatial error model (SEM) which assumes that the error term $\varepsilon$ from Eq. 1 does not meet the i.i.d conditions. The assumptions on the structural instability of the model (inconsistent estimators based on underlying processes) are shifted from the dependent variable to an unexplained impact in the error term. In the end, the error terms across spatial units are correlated. This is modelled by explicitly describing $\varepsilon$ as following a spatially autocorrelated process; the error term in the OLS model (Eq. 1) is then re-written as:

3 $$\varepsilon = \lambda W_{\varepsilon } + v$$

and so Eq. 1:

4 $$Y = X\beta + \lambda W_{\varepsilon } + v$$

where λ controls for heterogeneity in spatial autocorrelation (it will share the same properties as $\rho$ with values ranging from − 1 to + 1 to indicate the strength of the correlation). The weight matrix W would still be catching the geographical information from the SAR model but applied to the residual part of the estimation (identified now as $W_{\varepsilon }$). In the end, not accounting for the spatial error will lead to biased estimations and inefficient OLS.

The last spatial econometric model specifications model examined here is the spatial lag of X model (SLX), that focuses on the spillover effects between exogenous variables. $X\beta$ will be accompanied by a control vector Nx1 $WX\theta$ where the distance matrix will update the Kx1 vector of exogenous variables X. The $\theta$ will be a fixed Kx1 vector of unknown parameters. Factoring in all these to the OLS model in Eq. 1, it can be written as:

5 $$Y = X\beta + WX\theta + \varepsilon$$

The model specification in Eq. 5 will shift the weight of the spatial structure on the exogenous variables rather than the dependent variable *Y* or an unspecified process within the error term $\varepsilon$. It will account for the clustering effect within an area (a particular neighborhood and all the nearby ones). The number of gambling outlets in a neighborhood *i* would be dependent, not only on the socioeconomic and demographic conditions of the neighborhood, *X*, but also on the conditions of the surrounding area $\theta$.

A further theoretical assumption in all cases is that the number of gambling outlets within the borders of a certain urban area (neighborhood) reflects economic equilibrium, which is a standard assumption in the industrial organizational literature that studies firms’ entry into competitive and concentrated markets (Bresnahan & Reiss, 1990, 1991).

## Results

The previously described spatial econometric models are estimated by using maximum likelihood method in Stata 16 package. Variables not in percentages were transformed to their natural logarithms and an interaction term between income and unemployment is included (for ease of interpretation, both variables are mean-cantered). So estimated coefficients can be interpreted as elasticities as the sample mean.

Table 2 displays estimates from OLS (1), SAR (2), SEM (3), and SLX (4)–which includes the spatial lags of income. Results from a Wald test indicate that SEM model is preferred to others. The spatial parameter (λ) is positive and statistically significant (p-value 0.0056) confirming the result obtained from Moran’s I test. This indicates that, first, OLS predictors are insufficient to purge the spatial dependence in the outcome and, therefore the results from OLS regression would be misleading. Second, there is a positive significant spatial autocorrelation in this model which could mean that the feedback of the existing gambling market may favor the appearance of more gambling outlets (Economopoulos & Luxem, 2015).

**Table 2:** Determinants of gambling outlets location (neighborhoods of the city of Madrid. 2017)

	OLS(1)		SAR(2)		SEM(3)		SLX(4)	
	Coef	P > z	Coef	P > z	Coef	P > z	Coef	P > z
Income	− 0.511**	0.014	− 0.560	0.069	− 0.685**	0.014	− 0.690**	0.016
Unemployment	− 0.301	0.912	0.000	0.925	0.003	0.912	− 0.010	0.745
Unempl.*Income	0.995	0.069	1.001	0.104	1.030**	0.049	1.006	0.085
Population density	0.389***	0.000	0.391***	0.000	0.411***	0.000	0.391***	0.000
Native population	− 0.034	0.060	− 0.031	0.053	− 0.032**	0.050	− 0.038**	0.026
Youth population	− 0.049***	0.001	− 0.047***	0.000	− 0.044***	0.001	− 0.043***	0.001
Constant	8.837***	0.000	8.750***	0.001	10.209***	0.000	3.078	0.458
ρ			0.163	0.114				
λ					0.332***	0.006		
θ (Income)							0.748	0.086
Wald χ^2^ test			2.500	0.114	7.670***	0.006	2.940	0.086

131 Observations. **p < 0.05; ***p < 0.01. OLS (non-Spatial model); SAR = Spatial Autoregressive Regression model; SEM = Spatial Error Model; SLX = Spatial Lag of X model. The variables are in natural logarithmic form (except those expressed in percentage terms). Income and Unemployment rate are mean-centered

All thing considered, hereinafter, we comment on the results from SEM model. It should be noted that in SEM models there are not indirect effect, therefore, coefficients in Table 2 indicates total effects.

The results suggest that neighborhood household income have a negative, statistically significant effect on the number of gambling outlets located in such area. Concretely, a 1% increase in income is associated with a 0.68% decrease in the number of gambling outlets (at the sample data mean). This is in line with Welte et al. (2006) and Pearce et al. (2008), that confirm that the odds of gambling prevalence are higher when regions face lower income, and Dahan (2020), who suggests that the Israel National Lottery (Lotto) tend to set up significantly more sales points in disadvantaged neighborhoods, and it may suggest that the taxation of gambling outlets revenue is a regressive tax policy. Novak et al. (2006) reported a similar result for tobacco outlets. They found that retail tobacco outlets were disproportionately located in economic disadvantaged neighborhoods. In contrast, Espadafor and Martínez (2021), find no evidence of gambling outlets opening in already impoverished areas in the city of Madrid. However, it should be noted that they used no household income but rental price as an indicator of the area-level poverty. The negative sign of the estimated coefficient for income is consistent in all considered model specifications.

Evidence on the neighborhood unemployment is not statistically significant. This is consistent with some previous works, including Raisamo et al. (2019) that analyze location of electronic gambling machines (EGMs) in Finland providing evidence of insignificant effect of unemployment when combining all socio-economic indicators (income, unemployment, education) in the same model specification. However, the estimated effect of the interaction term between income and unemployment rate is positive and statistically significant (elasticity coefficient = 1.03). This means that the negative effect of household income on the number of gambling outlets is smaller in absolute value for urban areas with higher levels of unemployment. That is to say, the negative effect of income on the gambling retail environment is less negative as unemployment rate increases. This could be explained by some income inequality within a neighborhood that may attract gambling outlets due to an increase in propensity to gambling by lower-income citizens.

As for neighborhoods demographic characteristics, the estimated effect of population density population on the gambling retail environment is positive and statistically significant. A 1% increase in the density of the population, increases 0.41% the number of gambling outlets. This is an interesting result as it seems to contradict previous findings. Raisamo et al. (2019) found that the population density had no significance correlation with EGMs density. Also Wardle et al. (2014) suggested that it does not appear to be the case that areas with high concentrations of EGMs were those with a high density of people and jobs.

Regarding neighborhoods population structure, it is found that the ratio of native Spanish population negatively impacts the establishment of gambling outlets. This provides evidence contrary to Wenz (2008), who found that large numbers of native Americans in the county predict native American casino openings, but it is in line with multiple studies that reported that ethnic minorities may be at higher risk of developing gambling problems (see Welte et al., 2004, among others). Even in the case of other products whose consumption is linked to gambling (e.g. tobacco and alcohol), retail outlets were found to be more prevalent in neighborhoods with high concentrations of foreign-born residents (see Novak et al., 2006 and Bostean et al., 2021, among others). A similar result is obtained for the percentage of youth population. Percentage under age 18 is negatively associated with gambling density. Bostean et al. (2021) show a similar result for retail tobacco outlet but the opposite effect in the case of alcohol density.

All in all, results provide some evidence of co-location of gambling outlets within similar urban areas (neighborhoods). This confirms previous research showing co-location of individual types of outlets in similar geographical areas, including alcohol, fast food, tobacco, sub-prime financial services and even gambling outlet clusters (Kim, 2018; Macdonald et al., 2018; Pennay et al., 2021; Townshend, 2017).

## Discussion and Concluding Remarks

Gambling regulations worldwide aimed at expanding gambling opportunities and availability seemed to gradually make people more prone to gamble. Previous research has found a significant relationship between exposure to gambling, which is strongly influenced by the corresponding regulatory environment, and severe social concerns, including, among others, risk of gambling-related harm and/or regressivity of gambling taxation. Examining the spatial availability of gambling may provide a better understanding of the role of the retail environment in such social/public issues. This article focuses on the factors influencing recent expansion of gambling opportunities—i.e. the number of gambling outlets—within urban areas—i.e. neighborhoods. A number of interesting patterns are observed.

Living in neighborhood with low household income is linked to ease access to gambling opportunities. This is an interesting public finance finding, as may suggest that taxation of gambling business is a regressive tax policy (it should be acknowledged that, according to the nature of data and the study design, it cannot be certainly known that lower-citizens are those who exhibit a greater gambling prevalence). Also, resident population density positively impacts the establishment of gambling outlets, which interestingly seems to contradict previous findings. Finally, urban environments with older and/or non-native citizens host a higher number of gambling outlets. Overall, as shown by previous research on clusters of ‘environmental bads’ (alcohol, fast food, tobacco, gambling…), empirical spatial analysis demonstrates a strong correlation between neighborhood socio economic and demographic characteristics and access to gambling retailers highlighting a specific geographic patterning of distribution within more disadvantaged urban areas.

The results have interesting implications for gambling stakeholders and for local governments when it comes to the introduction and/or increase of gambling availability. In fact, this paper’s findings suggest that gambling opportunities display similar patterning and so the associated negative externalities may also have a spatial, geographical aspect, providing some support for policy measures to reduce concentration of gambling outlets in certain areas, such as low income neighborhoods—including restrictions on new outlets being opened, minimum distance requirements… All in all, understanding the distribution of gambling opportunities is an important public issue. In fact, public health concerns over gambling issues have been the strongest argument against the widespread expansion of gambling opportunities. This paper provides support of the need to regulate existing supply within the scope of the current regulatory framework. The overprovision of gambling outlets is a relevant urban policy matter, and so the findings here may be helpful in planning regulations appropriate for the urban areas in greatest need.

## Data Availability

Data is not available due to proprietary issues.

## Appendix

The first step in the construction of the spatial model is the definition of the relationship between the spatial units (neighborhoods). To do that, we construct a spatial weights matrix W. The matrix W will be a N × N symmetric, non-negative matrix of q-nearest neighbor value, attributing a higher value to the proximity of the spatial units. The principal diagonal will be 0 since it will indicate the neighborhoods distance with itself—the neighborhood cannot be a neighbor of itself.

To create a weights matrix we need to define what constitutes a neighbor. The condition to establish a relationship will be contiguity (sharing a border). The criteria under which the matrix W will establish a contiguity relationship can be rook or queen. Rook, more restrictive will only consider two spatial units to be neighbors if there is a common border between the two. Contiguity criterion queen for the weights matrix will be more inclusive, taking vertexes into account when establishing a connection. Because of this, in this paper we have considered the queen assumption. The contiguity criterion gives a non-zero value to spatial units that are considered neighbors but it doesn’t dismiss the relationship between non-neighboring spatial units.

For N spatial units, the weights matrix will be defined as:

6 $$W = \left( {\begin{array}{*{20}c} {W_{11} } & {W_{12} } & {\begin{array}{*{20}c} \ldots & {W_{1n} } \\ \end{array} } \\ {W_{21} } & {W_{22} } & {\begin{array}{*{20}c} \ldots & {W_{n2} } \\ \end{array} } \\ {\begin{array}{*{20}c} \ldots \\ {W_{n1} } \\ \end{array} } & {\begin{array}{*{20}c} \ldots \\ {W_{n2} } \\ \end{array} } & {\begin{array}{*{20}c} {\begin{array}{*{20}c} \ldots & \ldots \\ \end{array} } \\ {\begin{array}{*{20}c} \ldots & {W_{nn} } \\ \end{array} } \\ \end{array} } \\ \end{array} } \right)$$

Prior to the estimation of the model, the weights matrix W is row normalized (but not column normalized). The sum of the elements of each row will be 1.

For each $W_{ij}$ with $i \ne j$:

7 $$W_{ij} = \frac{{W_{ij} }}{{\mathop \sum \nolimits_{j = 1}^{N} W_{ij} }}$$

The row and column implications of the weights matrix are as follows: the row elements of the weights matrix show the impacts on a spatial unit from the rest of each spatial unit; meanwhile the column elements of a weights matrix display the impact of a spatial unit on all others (the inverse effect). For this reason and as mentioned before, the specification of the principal diagonal of the weights matrix has to be 0—a neighborhood cannot have an impact on itself since it would create a heteroscedasticity problem. For all N neighborhoods of the sample, $W_{nn}$ will always be 0.
